# FACTORS ASSOCIATED WITH EATING PERFORMANCE IN NURSING HOME RESIDENTS WITH DEMENTIA AND MULTIPLE COMORBIDITIES

**DOI:** 10.1093/geroni/igac059.1076

**Published:** 2022-12-20

**Authors:** Wen Liu, Elizabeth Galik, Barbara Resnick

**Affiliations:** University of Iowa, Iowa City, Iowa, United States; University of Maryland, Baltimore, Baltimore, Maryland, United States; University of Maryland, Baltimore, Maryland, United States

## Abstract

Eating performance is the functional ability to get food and drink into the mouth. Nursing home residents with dementia commonly experience compromised eating performance and subsequent nutritional consequences. Resident characteristics including cognition, physical capacity, and functional ability are associated with eating performance. This study aimed to test the association between functional ability, behavioral symptoms, psychotropic medication use, and comorbidities and eating performance. This was a secondary analysis using baseline data from two randomized controlled trials testing the impact of Function Focused Care on function and behavioral symptoms between 2014-2020. A total of 889 residents with moderate-to-severe dementia (mean age 86.58 years, 72% female, 70% white, 69.5% severe dementia) from 67 nursing homes in two states were recruited. Eating performance (dependent variable) was measured using the single self-feeding item on the Barthel Index. Independent variables included functional ability (Barthel Index total score excluding self-feeding item score), behavioral symptoms (agitation, depression, resistiveness to care), and psychotropic medication use (anti-depression, sedative, anti-psychotics, anti-seizure, anti-anxiety), and comorbidities. Residents had on average 5 documented comorbidities (SD=3.06, range=0-12) and were on approximately 1 psychotropic medication (range=0-5, SD=1.24). Thirty-eight percent of residents were dependent in eating performance. Functional ability (OR=1.052, 95% CI=1.043,1.061, p<.001), depression(OR=.931, 95% CI=.887,.978, p=.004), and anti-anxiety medication use(OR=.632, 95% CI=.409, .978, p=.039) were associated with eating performance. Findings supported better functional ability, lower depression, and less anti-anxiety medication use were associated with better eating performance. Targeted efforts including maintaining functional ability, minimizing anti-anxiety medication use, and managing depression are encouraged to support eating performance.

